# URBAN–RURAL DISPARITIES IN CHRONIC DISEASE PROGRESSION AMONG CHINESE OLDER ADULTS

**DOI:** 10.1093/geroni/igad104.2542

**Published:** 2023-12-21

**Authors:** Yuanchang Zhao, Jessica Kelley

**Affiliations:** Case Western Reserve University, Cleveland, Ohio, United States; Case Western Reserve University, Cleveland, Ohio, United States

## Abstract

Over the last thirty years, China has experienced rapid increases in the prevalence of hypertension and diabetes, across urban and rural areas. Studies do indicate that the prevalence rates both hypertension and diabetes are higher in urban areas than in rural areas, seeming to indicate higher chronic disease burden among urban older adults. Few studies, however, focus on the progression of hypertension and diabetes once diagnosed. Given the likelihood of under-diagnosis in rural areas, the aim of our study is to examine urban-rural disparities in the progression of hypertension and diabetes among older Chinese who have been diagnosed with either or both conditions. We utilize two waves of the CHARLS, examining adults over age 50 (n=5,067). Our findings confirm that rural older adults are less likely to be diagnosed with diabetes or hypertension than urban older adults, net of other risk factors. Among those with a diagnosis, and controlling for whether they received treatment, the nested logistic regression models indicate that rural older adults report 32% higher odds of worsening hypertension compared to their urban counterparts. Likewise, the odds of worsening diabetes are 52% higher in rural older adults than in urban older adults. Our findings indicate that although rural older adults are less likely to have a diagnosis of diabetes or hypertension, among those with a diagnosis, rural older adults tend to be sicker and worsen more quickly. We discuss the implications of rural-urban disparities in access to health care and treatment.

